# The Pocket Materia Medica and Therapeutics

**Published:** 1891-08

**Authors:** 


### Book Reviews.

THE POCKET MATERIA MEDICA AND THERAPEUTICS. C. Henri Leonard, A. M., M. D., 1891: The Illustrated Medical Journal Co., Detroit, Mich.

However familiar the practitioner may be with the substances and compounds touched upon from daily experience with the employment of drugs, etc., he has, as a rule, only a partial knowledge of their respective origin, formula, mode of preparation, and often but a nebulous conception of their therapeutic value and adaptability to pathological conditions.

This little book, the production of four years' research and arduous labor, evinces ability and a mind completely "*au fait*" of the subjects it treats.

Although a *resume* of actions and doses, it is a valuable multum in parvo reference for the physician and unquestionably an aid to the beginner and inexperienced. E. VAN G.

